# Supplementary material for: Curated and harmonised transcriptomics datasets of interstitial lung diseases
Source: Data Brief. 2025 Oct 14;63:112139. doi: 10.1016/j.dib.2025.112139 (PMC12581653; doi:10.1016/j.dib.2025.112139)
Supplement: Supplementary file 1 [file mmc1.zip › Supplementary_material/RNA-seq/fastQC reports/GSE180415/TRIMMED_SRR15194701_2_fastqc.html]

TRIMMED\_SRR15194701\_2.fastq.gz FastQC Report 

FastQC Report

Wed 25 Oct 2023  
TRIMMED\_SRR15194701\_2.fastq.gz

## Summary

- Basic Statistics
- Per base sequence quality
- Per sequence quality scores
- Per base sequence content
- Per sequence GC content
- Per base N content
- Sequence Length Distribution
- Sequence Duplication Levels
- Overrepresented sequences
- Adapter Content

## Basic Statistics

| Measure | Value |
| --- | --- |
| Filename | TRIMMED\_SRR15194701\_2.fastq.gz |
| File type | Conventional base calls |
| Encoding | Sanger / Illumina 1.9 |
| Total Sequences | 38359596 |
| Sequences flagged as poor quality | 0 |
| Sequence length | 60-80 |
| %GC | 55 |

## Per base sequence quality

## Per sequence quality scores

## Per base sequence content

## Per sequence GC content

## Per base N content

## Sequence Length Distribution

## Sequence Duplication Levels

## Overrepresented sequences

| Sequence | Count | Percentage | Possible Source |
| --- | --- | --- | --- |
| GGTGGCGCGTGCCTGTAGTCCCAGCTACTCGGGAGGCTGAGGCTGGAGGA | 76062 | 0.1982867598501298 | No Hit |
| GATCAATCGCCCCCGGGGGTGCCTCCGGGCTCCTCGGGGTGCGCGGCTGG | 70240 | 0.18310933201694826 | No Hit |
| GGGGGGGGGGGGGGGGGGGGGGGGGGGGGGGGGGGGGGGGGGGGGGGGGG | 68819 | 0.17940491344069423 | No Hit |
| GGTGGCGCGTGCCTGTAGTCCCAGCTACTCGGGAGGCTGAGGTGGGAGGA | 66663 | 0.17378441629051566 | No Hit |
| GCGGTGGCGCGTGCCTGTAGTCCCAGCTACTCGGGAGGCTGAGGCTGGAG | 57386 | 0.14960011570507675 | No Hit |
| GTTCTGGGCTGTAGTGCGCTATGCCGATCGGGTGTCCGCACTAAGTTCGG | 57344 | 0.14949062550085251 | No Hit |
| CGGTGGCGCGTGCCTGTAGTCCCAGCTACTCGGGAGGCTGAGGCTGGAGG | 54282 | 0.14150826823097928 | No Hit |
| CCGGGCTCCTCGGGGTGCGCGGCTGGGGGTTCCCTCGCAGGGCCCGCCGG | 51097 | 0.13320526107730646 | No Hit |
| GCGGTGGCGCGTGCCTGTAGTCCCAGCTACTCGGGAGGCTGAGGTGGGAG | 49077 | 0.12793930363604455 | No Hit |
| CGGTGGCGCGTGCCTGTAGTCCCAGCTACTCGGGAGGCTGAGGTGGGAGG | 48363 | 0.12607797016423217 | No Hit |
| TTTCTATAGTGTGTTACTAGAGAAGTTTCTCTGAACGTGTAGAGCACCGA | 39282 | 0.10240462386517314 | No Hit |

## Adapter Content

Produced by FastQC (version 0.11.7)
